# Mixing Online and Face-to-Face Therapy: How to Benefit From Blended Care in Mental Health Care

**DOI:** 10.2196/mental.4534

**Published:** 2016-02-09

**Authors:** Jobke Wentzel, Rosalie van der Vaart, Ernst T Bohlmeijer, Julia E W C van Gemert-Pijnen

**Affiliations:** ^1^ Center for eHealth and Wellbeing Research Department of Psychology, Health and Technology University of Twente Enschede Netherlands; ^2^ Unit of Health, Medical and Neuropsychology Leiden University Leiden Netherlands

**Keywords:** blended care, Internet-delivered cognitive behavior therapy, mental health care, online, shared decision making

## Abstract

Blended care, a combination of online and face-to-face therapy, is increasingly being applied in mental health care to obtain optimal benefit from the advantages these two treatment modalities have. Promising results have been reported, but a variety in descriptions and ways of operationalizing blended care exists. Currently, what type of “blend” works for whom, and why, is unclear. Furthermore, a rationale for setting up blended care is often lacking. In this viewpoint paper, we describe postulates for blended care and provide an instrument (Fit for Blended Care) that aims to assist therapists and patients whether and how to set up blended care treatment. A review of the literature, two focus groups (n=5 and n=5), interviews with therapists (n=14), and interviews with clients (n=2) were conducted to develop postulates of eHealth and blended care and an instrument to assist therapists and clients in setting up optimal blended care. Important postulates for blended care are the notion that both treatment modalities should complement each other and that set up of blended treatment should be based on shared decision making between patient and therapist. The “Fit for Blended Care” instrument is presented which addresses the following relevant themes: possible barriers to receiving blended treatment such as the risk of crisis, issues in communication (at a distance), as well as possible facilitators such as social support. More research into the reasons why and for whom blended care works is needed. To benefit from blended care, face-to-face and online care should be combined in such way that the potentials of both treatment modalities are used optimally, depending on patient abilities, needs, and preferences. To facilitate the process of setting up a personalized blended treatment, the Fit for Blended Care instrument can be used. By applying this approach in research and practice, more insight into the working mechanisms and optimal (personal) “blends” of online and face-to-face therapy becomes within reach.

##  Introduction

The use of eHealth has shown promising results in various mental health treatments [1], especially when guidance from a care provider is included [2-4]. eHealth also provides opportunities for self-management and continuity of care. This combination of advantages of online and offline guidance and treatment render positive outcomes, making it a good alternative to regular face-to-face treatment [5].

In recent studies, this care provider guidance is mostly offered through email. Still, in order to offer patients the “best of both worlds,” the use of *blended* treatments, in which a combination of online (or mobile) components and face-to-face components is applied, is rising in clinical practice as well. Blended treatment or blended care is described in literature as “technology-supported care.” However, a clear definition of what blended care precisely withholds is currently unavailable, because blended care is operationalized in various ways. Studies have been performed on computer-assisted therapy as a partial replacement of face-to-face sessions (sessions become shorter, not less frequent) [6]; unguided self-help modules combined with (scripted) face-to-face sessions to reflect on the progress of online treatment programs with a therapist [7]; and online modules as an addition to face-to-face depression treatment sessions [8]. Literature shows that blended care treatment can offer synchronous or asynchronous guidance, support via online guidance or self-help modules, and can apply personal or automated feedback and support, with promising effects [9]. Nevertheless, studies have shown that online and face-to-face treatments are often not integrated, but rather online components are used as an addition to regular therapy [3,10,11]. Moreover, often no rationale for applying specific blends of online and face-to-face treatment components is provided. These descriptions reflect the role of technology as supportive to traditional treatment, and do not acknowledge the potential equal contribution of both modalities of care.

To define blended mental health treatment and to decide which face-to-face components can be replaced by online modules is difficult. Up until now, only a fraction of all possible applications of blended treatment in mental health care has been explored and described. Previous research shows that there is a lack of knowledge on what exactly constitutes blended care, who can benefit from it, and how blended treatments should be set up [12]. This lack of research into the “ingredients” of proper blended care makes it hard to determine the effectiveness of this form of treatment and to advance implementation of blended care in practice. More specifically, questions like “what works for whom, and in what blend?” need to be answered. Therefore, the aim of this article is to describe postulates for blended care and to propose a strategy to implement blended care in a clinical setting based on predictors of successful online treatment that can be assessed previous to or during treatment.

Our viewpoint and postulates are based on previous research on the uptake of blended care and implementation of eHealth applications and e(mental) care in secondary care practice [12-14]. In a former study, we investigated barriers and facilitators to blended care [12]. First, we considered studies on types (operationalization) of blended care, and asked patients and therapists what their preferences for certain blended treatment configuration are, in a Delphi study [12]. These results showed that patients and therapists differ in their preference for division of online and face-to-face components. Besides, therapist did not have clear ideas on how online treatment can support face-to-face treatment; they asked for a guidance on how to blend online and offline care. This shows that adequate information and discussions between patient and therapist regarding the treatment operationalization are essential to enhance understanding and agreement on proper treatment. Further, therapists reported that the complexity of patients’ problems calls for a tailored blended treatment. What parts of treatment can best be offered online or face-to-face can differ between patients (based on ability, preference, severity, and type of problems) and should thus be considered for each patient individually. These findings indicate that blended treatment is no fixed formula, and should be approached as an opportunity to integrate treatment modalities to reach a proper, tailored treatment plan. We describe the postulates for blended care that describe how blended care can fulfill this goal.

##  Postulates for Blended Care

To propose a definition of blended care, in which online and face-to-face components are used to their fullest potential to create an optimal combination, we defined the following postulates, based on our research in eHealth and blended care [12,13]:

1. The term “blended” refers to an integration of online and offline components in a treatment process. This means that online and offline components are interconnected in some way and not standalone treatment pathways [12].
2. Both the technology and face-to-face modalities contribute substantively and procedurally to the treatment process. This means that the use of online components contributes equally to the therapy as face-to-face components do [12].
3. In addition, online components should be carefully selected and adjusted to the treatment process and progress. This means that a standard 50:50 ratio of online and face-to-face care does not (always) suffice. Rather, weighing reasons and carefully deciding to apply one modality or the other are called for, while keeping a close look on the interrelatedness of both treatment modalities [12].
4. The integration of offline and online components should be based on the protocol for treatment, the capacities of technology to motivate and support patients to follow the treatment process, and the characteristics and capabilities of patients to receive and participate in online treatment [13]. This means that blended care is dynamic and flexible, as technology has the capacity to present the content in a nonlinear and dynamic way using text, images, interactive assignments, etc. Furthermore, it enables monitoring of online activities to intervene in an early stage when needed [13].
5. Therapists must consider the rationale for providing face-to-face and/or online modalities, following discussions with the patient to assure the fit between technology and the end users [13].

With these postulates in mind, a rationale for applying, developing, and researching blended mental health care is provided.

##  What Is Needed to Benefit From Blended Mental Health Treatment?

To translate these postulates into an instrument that summarizes relevant considerations in setting up blended treatment and guides therapists and clients through the process of jointly discussing and setting up blended treatment, we performed an additional literature search aimed at identifying predictors of blended treatment success. Only by knowing what variables play a role in desirable reach, use, and adherence of online therapy, the fit between a patient and a combination of online and face-to-face therapy can be created. A literature search on predictors of successful online treatment for depression showed that various variables play a part in the process of therapy use, adherence, and success (for a complete overview, see Multimedia Appendix 1). First of all, people who have access to certain practical resources, are able to benefit from it. These resources include Internet access [15,16], a computer [15,16], and a place to work in safety and privacy [16]. In addition, having experience with computers and the Internet [16] and sufficient eHealth literacy [15,16] are necessary for online mental health treatment. Having enough time to integrate the treatment into (daily) life routine also facilitates online care [17,18]. Social resources are important for a successful treatment as well: support from a partner (or someone in the immediate vicinity of the patient) can improve discipline to use eHealth [16,17]. On a personal level, motivation and willingness to complete online therapy [17-20], trust in and credibility of the therapy [17,20-22], and need for support [17] during therapy all contribute to successful blended treatment. Independency, being disciplined, and being able to work in a structured way are also influential to treatment success [20]. Finally, personality traits have been reported to be associated with online treatment outcomes, as well as with locus of control, self-determination, and commitment and involvement in therapy [23-26]. Overall, these findings show that besides the practical necessities such as having a computer and Internet access, most predictors are facilitators rather than prerequisites for blended treatment, when treatment can be attuned to these particular characteristics.

To validate these facilitators for blended care, we invited health care professionals to discuss the rationale for the development of the instrument. We conducted a focus group in which 5 therapists (3 males) participated. These therapists are (mainly) experienced in treating patients with mood and anxiety disorders, personality disorders, and have (self-proclaimed) previous experience with eMental health treatment. In addition, we held individual interviews with 14 therapists (7 males), who had varying levels of experience in online or blended treatment. These clinical psychologists are (mainly) experienced in treating patients with mood and anxiety disorders, personality disorders, and developmental disorders. One of these therapists worked as a manager. These consultations with secondary health care therapists were essential to apply a practice-driven scope to the predictors we identified in literature. The focus of these iterative consultations was to assess how the predictors can be used in practice to help predict and anticipate blended treatment success. These discussions revealed that in practice, very few criteria make people fully unfit to benefit from blended mental health treatment: presence of practical barriers (no computer or Internet access, no place to work) and insufficient (cognitive) skills (intelligence quotient and Internet skills that do not match the program’s minimal requirements). Rather, therapists claim that adjusting the treatment to a person’s specific situation, needs, and abilities is an important predictor of treatment success. This is in line with what was found in our earlier study among both therapists and patients, that is, a discussion between patient and therapist is essential [12].

Therefore, to facilitate this dialogue on how to jointly decide on the configuration of the blended treatment, we created a shared decision-making instrument (Fit for Blended Care). This instrument addresses the topics regarding the needs, characteristics, and skills of an individual that need to be discussed to enable therapists and patients to decide in which way blended therapy can best be applied. During the developmental process of the instrument, formative evaluations [13] were conducted with follow-up focus groups consisting of 5 therapists. One of these participants is male; all therapists are experienced in treating patients with substance-related disorders and in eMental health treatment. In addition, 2 clients (both treated for personality disorder; one of them is male) were interviewed. The interviews and focus groups were done to cocreate and assure a fit between the content of the instrument and actual practice. These consultations provided input on formulation on the questions, and also on how such content and system would preferably be applied by care providers in clinical practice. This led to the insight that strict formulation of topics (by providing checklist rules; “yes/no” answers) leaves too little room for interpretation of the specific situation of the patient. Issues with specific items (in what situation would crisis or suicidality actually be a risk for starting or receiving blended treatment) continued to surface in our discussions, stressing the need for therapists to make their own assessment of the risks and discussing these with the patient and documenting the outcome of the discussion. In conclusion, care providers preferred checklist topics to start a shared decision-making process with their patients on the use and distribution of online care components.

### An Instrument for Implementation in Practice

To support therapists and patients in outlining a fitting blended treatment, we created an instrument to assist a guided dialogue between therapists and patients, which is needed to shape and set up the blended treatment in such way that it matches patient characteristics (including abilities, needs, and preferences), according to our postulates, prior experiences with blended care [12], and underlying eHealth approach [13]. The aim of the instrument is to provide input for a conversation that leads to shared decision making on blended treatment setup, and creates awareness among both patient and therapist regarding issues that are relevant to blended treatment success. Because of the (preferably) natural course of such shared decision making, no checklist rules are provided. Rather, the topics that need to be discussed or those that patient and therapist should be aware of are summarized. This way, the topics (concepts) can be operationalized by therapists to match their own working definitions.

The “Fit for Blended Care” instrument consists of four main parts: (1) *prerequisites*, which are items on (mostly practical) preconditions that need to be met to be able to start blended treatment (9 items); (2) *possible barriers*, which are items on issues that might hinder blended treatment (5 items); (3) *possible facilitators*, which are items on issues that can facilitate blended treatment, and should be considered when deciding to start a blended treatment (6 items). (4) *advice overview*, an (written) overview of the possible barriers and facilitators that prompts therapists and patients to discuss and decide on the composition of blended treatment. Every item (barriers and facilitators) is linked to a specific advice, which can be considered if the item proved to be relevant (based on the answers and discussion). The advice describes how to deal with facilitators and barriers to blended treatment. Furthermore, it provides suggestions for additional agreements on what to do if a problem related to barriers presents itself. The therapist and patient discuss these outcomes prior to the start of treatment and use the discussion to make agreements on what type of blended care the patient receives. Some of the items can be answered in advance by the therapist or by the patient (eg, before or during intake) to facilitate and speed up the process. Currently, the full content of the instrument (items and advice) is available in Dutch [27]. Textbox 1 provides an overview of the instrument items in English. An additional rationale per item is included in Multimedia Appendix 2.

**Fit for Blended Care instrument overview (in English).**

Therapist checks

1.1. Are appropriate online modules available related to the main symptoms/diagnosis of the patient?

1.2. Is there absence of (current) crisis (eg, severe suicidality or psychotic symptoms)?

1.3. Is there absence of an acute medical care need (that may hinder the patient’s ability to independently work on his/her treatment?)

1.4. Is the patient’s intelligence quotient match sufficient for the blended treatment content?

Patient checks

1.5. Does he/she have computer access?

1.6. Does he/she have Internet access?

1.7. Does he/she have a private, safe place to work?

1.8. Does he/she have sufficient Internet skills?

Patient and therapist discussions:

1.9. Does the patient have sufficient writing (expression) skills?

2.1. The patient’s motivation and trust

2.2. The patient’s risk of crisis

2.3. Cognitive problems that may hinder treatment

2.4. Psychosocial problems that may hinder treatment

2.5. Other issues/comorbidity that may hinder treatment

3.1. Whether they have (or chances on having) a good therapeutic/working relation

3.2. Practical reasons for preferring blended care, e.g., saving on cost and time, comfort.

3.3. Possible other reasons for preferring blended care such as stigma or safety issues (shame of having to enter a clinic, discussing reason for taking time off work with employer, fears of going out into public to travel to a clinic)

3.4. The likeliness of being able to be open in online communication

3.5. Is the patient conscientious?

3.6. Does the patient have a social support network?

## Implications for Research and Practice

Blended mental health care may have some important advantages over face-to-face therapy. The client is encouraged to continue his or her treatment between the sessions with the therapist in a structured way. Likewise, blended care has advantages over online therapy because it enables personal guidance (face-to-face) when needed, and possibly a better adherence to treatment. This may make blended care treatments more cost-effective than face-to-face therapies. Moreover, the possibility to work on their mental health between sessions encourages clients’ trust in their own abilities to self-manage and adapt, which are defined as core aspects of health [28]. These skills are supported by and united with blended treatment programs. Within a context of a large number of people suffering from mental health problems and limited professional resources, blended mental health care may offer treatment modalities that are both effective and affordable. However, more research and innovation is warranted to decide what blend is preferred by clients and therapists in certain situations.

### Implications for Research

A rapidly growing number of meta-analyses demonstrate the efficacy of both face-to-face and online treatments for psychological disorders [29,30]. However, the implications of using technology to support online treatments have hardly been studied yet. It is recommended to compare blended care treatments with current state-of-the-art, face-to-face treatments, to study whether similar effects can be obtained at lower costs and with similar client satisfaction. In addition, attention should be paid to understand which form blended care is effective and why, not overlooking the special role technology design has in such studies.

At present, little is known about this. Therefore, we have created an instrument that supports decision making in the preferred format for blended treatment. The effects of using this instrument and the underlying motivations and mechanisms on decision making should be studied.

### Implications for Practice

Blended mental health care is increasingly being applied and therapists and patients are discovering the opportunities of adding technology to treatment. The use of information and communication technology inherently calls for personalization of care; it offers a multitude of possibilities for tailored, personal treatment. This process of adjusting the design and content of treatment to patient (and therapist) needs and preferences is facilitated by technology. However, not much is known about why certain “blends” of design and content are chosen and applied, and with what rationale. Based on experiences from practice (best practices), and the postulates and instrument we provide, a well-thought rationale for blended care can be applied. The use of the postulates can support a therapist’s or organization’s own approach to blended care, and likewise, the instrument may facilitate implementation and actual execution of blended treatment. It was created to support practice and create awareness about topics relevant for (starting) blended treatment.

### Conclusions

Blended care offers new possibilities in terms of personalized mental health care treatment. Technology can (at least partially) replace face-to-face contact. Blended care invites patients and therapists to think about personal needs and preferences, for an optimally personalized treatment that can enhance the self-management of patients and translation of treatment into daily life. However, to reach the full potential of blended care, more insight is needed into what suits whom and how technology features and treatment operationalization via technology can be optimized. These fundamental issues should be of primary concern, as barriers for implementation and adherence lie within technology and organization of health care, not in specific patients or patient profiles [13]. We invite scholars to discuss our findings and ideas and to explore the underlying mechanisms that explain why blended care is of added value, in what format and to whom.

## Appendix

Multimedia Appendix 1 Literature review outcomes overview.

Multimedia Appendix 2 Instrument.

## References

[1] Richards D, Richardson T (2012). Computer-based psychological treatments for depression: A systematic review and meta-analysis. Clin Psychol Rev.

[2] Fledderus M, Bohlmeijer E, Pots Wendy T M, Meulenbeek Peter A M, Ten Klooster Peter M, Schreurs Karlein M G (2016). Acceptance and commitment therapy as a web-based intervention for depressive symptoms: randomised controlled trial. Br J Psychiatry.

[3] Meglic M, Furlan M, Kuzmanic M, Kozel D, Baraga D, Kuhar I, Kosir B, Iljaz R, Novak SB, Dernovsek MZ, Marusic A, Eysenbach G, Brodnik A (2010). Feasibility of an eHealth service to support collaborative depression care: Results of a pilot study. J Med Internet Res.

[4] Andersson G, Cuijpers P, Carlbring P, Riper H, Hedman E (2014). Guided Internet-based vs. face-to-face cognitive behavior therapy for psychiatric and somatic disorders: A systematic review and meta-analysis. World Psychiatry.

[5] Månsson KNT, Skagius RE, Gervind E, Dahlin M, Andersson G (2013). Development and initial evaluation of an Internet-based support system for face-to-face cognitive behavior therapy: A proof of concept study. J Med Internet Res.

[6] Wright J, Wright A, Salmon P, Kuykendall J, Goldsmith L, Zickel M (2002). Development and initial testing of a multimedia program for comptuer-assisted cognitive therapy. Am J Psychother.

[7] Wilhelmsen M, Lillevoll K, Risør M, Høifødt R, Johansen M, Waterloo K, Eisemann M, Kolstrup N (2013). Motivation to persist with internet-based cognitive behavioural treatment using blended care: A qualitative study. BMC Psychiatry.

[8] Krieger T, Meyer B, Sude K, Urech A, Maercker A, Berger T (2014). Evaluating an e-mental health program ("deprexis") as adjunctive treatment tool in psychotherapy for depression: Design of a pragmatic randomized controlled trial. BMC Psychiatry.

[9] Kelders SM, Bohlmeijer ET, Pots WTM, van Gemert-Pijnen JEWC (2015). Comparing human and automated support for depression: Fractional factorial randomized controlled trial. Behav Res Ther.

[10] Meyer B, Berger T, Caspar F, Beevers CG, Andersson G, Weiss M (2009). Effectiveness of a novel integrative online treatment for depression (Deprexis): Randomized controlled trial. J Med Internet Res.

[11] Robertson L, Smith M, Castle D, Tannenbaum D (2006). Using the Internet to enhance the treatment of depression. Australas Psychiatry.

[12] van der Vaart R, Witting M, Riper H, Kooistra L, Bohlmeijer ET, van Gemert-Pijnen LJ (2014). Blending online therapy into regular face-to-face therapy for depression: Content, ratio and preconditions according to patients and therapists using a Delphi study. BMC Psychiatry.

[13] van Gemert-Pijnen JEWC, Nijland N, van LM, Ossebaard HC, Kelders SM, Eysenbach G, Seydel ER (2011). A holistic framework to improve the uptake and impact of eHealth technologies. J Med Internet Res.

[14] Van Gemert-Pijnen JEWC, Peters O, Ossebaard HC (2013). Improving eHealth.

[15] Waller R, Gilbody S (2009). Barriers to the uptake of computerized cognitive behavioural therapy: A systematic review of the quantitative and qualitative evidence. Psychol Med.

[16] Gerhards SAH, Abma TA, Arntz A, de Graaf LE, Evers SMAA, Huibers MJH, Widdershoven GAM (2011). Improving adherence and effectiveness of computerised cognitive behavioural therapy without support for depression: A qualitative study on patient experiences. J Affect Disord.

[17] Donkin L, Glozier N (2012). Motivators and motivations to persist with online psychological interventions: A qualitative study of treatment completers. J Med Internet Res.

[18] Farrer LM, Griffiths KM, Christensen H, Mackinnon AJ, Batterham PJ (2013). Predictors of adherence and outcome in Internet-based cognitive behavior therapy delivered in a telephone counseling setting. Cogn Ther Res.

[19] Neil AL, Batterham P, Christensen H, Bennett K, Griffiths KM (2009). Predictors of adherence by adolescents to a cognitive behavior therapy website in school and community-based settings. J Med Internet Res.

[20] Bendelin N, Hesser H, Dahl J, Carlbring P, Nelson KZ, Andersson G (2011). Experiences of guided Internet-based cognitive-behavioural treatment for depression: A qualitative study. BMC Psychiatry.

[21] Nordgreen T, Havik OE, Ost LG, Furmark T, Carlbring P, Andersson G (2012). Outcome predictors in guided and unguided self-help for social anxiety disorder. Behav Res Ther.

[22] Cavanagh K, Shapiro DA, Van Den Berg S, Swain S, Barkham M, Proudfoot J (2009). The acceptability of computer-aided cognitive behavioural therapy: A pragmatic study. Cogn Behav Ther.

[23] Wojtowicz M, Day V, McGrath PJ (2013). Predictors of participant retention in a guided online self-help program for university students: Prospective cohort study. J Med Internet Res.

[24] MacLeod M, Martinez R, Williams C (2009). Cognitive behaviour therapy self-help: Who does it help and what are its drawbacks?. Behav Cogn Psychother.

[25] Christensen Helen, Griffiths Kathleen M, Farrer Louise (2009). Adherence in internet interventions for anxiety and depression. J Med Internet Res.

[26] Mohr DC, Cuijpers P, Lehman K (2011). Supportive accountability: A model for providing human support to enhance adherence to eHealth interventions. J Med Internet Res.

[27] Universiteit Twente (2015). Fit for Blended Care.

[28] Huber M, Knottnerus JA, Green L, Horst HVD, Jadad AR, Kromhout D, Leonard B, Lorig K, Loureiro MI, Meer JWMVD, Schnabel P, Smith R, Weel CV, Smid H (2011). How should we define health?. BMJ.

[29] Veehof MM, Oskam M, Schreurs KMG, Bohlmeijer ET (2011). Acceptance-based interventions for the treatment of chronic pain: A systematic review and meta-analysis. Pain.

[30] Cuijpers P, Dekker J (2005). Psychological treatment of depression: A systematic review of meta-analyses. Ned Tijdschr Geneeskd.

