# Supplementary material for: Mixing Online and Face-to-Face Therapy: How to Benefit From Blended Care in Mental Health Care
Source: JMIR Ment Health. 2016 Feb 9;3(1):e9. doi: 10.2196/mental.4534 (PMC4764785; doi:10.2196/mental.4534)
Supplement: Multimedia Appendix 1 [file mental_v3i1e9_app1.pdf]

Developing an instrument to predict treatment success in blended care for depression: an overview of predictors found in literature

Table 1. Studies that address predictors, determinants, and motivators for use, acceptance, or effect of online therapy for depression or fear.

Article	Type of intervention and target group	Study type	Determinants or predictors under study	Outcomes
[1] Batterham, 2008 (AUS)	Online self-help CBT for depression; MoodGYM<br>Duration: 5 weeks<br>Target group: community	Logistic regression to identify predictors of adherence	▪ Demographics<br>▪ Severity of depression<br>▪ Disfunctional thinking	▪ Younger participants had a better adherence<br>▪ Higher educated patients had a better adherence<br>(authors suggest that these (above) findings might be due to internet-skills; were not measured)<br>▪ Referral by health professional and more severe complaints are associated with better adherence<br>▪ Higher levels of 'disfunctional thinking' are associated with better adherence
[2] Bendelin, 2011 (SWE)	Online self-help CBT for depression with minimal therapist contact<br>Duration: 8 weeks<br>Target group: General public; mild to moderate symptoms	Qualitative study with patient (user) interviews	From the thematic analysis themes emerged: werk process, motivation, attitude consequences.<br><br>Division of patients into "readers", "strivers" and "doers" can be made.	Barriers:<br>▪ Difficulty bringing theoretical (reading) content into practice (readers)<br>▪ Difficulty deciding on workload and pace<br>▪ Lack of support/extrinsic motivator<br>▪ Sceptisism about online therapy<br>Motivators:<br>▪ No difficulties bringin theoretical (reading content into practice (doers)<br>▪ Structured way of working<br>▪ Indepencence
[3] Cavanagh, 2009 (UK)	Beating the blues; CCBT program consisting of clinic visits with short face-to-face support meetings<br>Duration: 8 sessies<br>Target group: patients referred	Evaluation of the program via questionnaires. Between-group differences are analyzed with t-tests	Predictors of program completion and effect:<br>Demographics<br>Computer experience<br>CBT credibility<br>Attitudes to CBT	Only treatment credibility en positive expectations of CCBT predicted treatment completion

	by GP or community health care professionals.			
[4] Donkin, 2012 (AUS)	e-Couch: self-help CBT intervention for cardiovascular risk patients suffering from depression.<br>Duration: 12 weeks<br>Target group: cardiovascular risk patients	Qualitative study by means of patient (user) interviews	From thematic analyses barriers and motivators emerged	Important barriers that were found are:<br>▪ Time constraints<br>▪ Competing priorities/forgetting it<br>▪ Problems regarding mood and anxiety<br>▪ Computer 'frustrations'<br>▪ Lack of trust in therapeutic relationship via computer/lack of personalisation<br>Important motivators are:<br>▪ Intrinsic motivation/persistence<br>▪ Sense of control/work in own time and pace<br>▪ Creating a habit/daily routine<br>▪ Identification with the program
[5] Farrer, 2014 (AUS)	MoodGYM: Self-adminstered psychoeducation and CBT intervention (web only and web with tracking)<br>Duration: 6 weeks<br>Target group: callers to a national crisis telephone counseling service	Linear regression analysis<br>+ extra assessment of reasons for dropout among 10 participants	▪ Demographic variables (age, sex, education, marital status, employment)<br>▪ Baseline depression severity<br>▪ Motivation (NML-P)	Better adherence with:<br>▪ Higher levels of education<br>▪ Higher levels of motivation<br>▪ younger age<br>▪ lower levels of baseline depression<br>Extra assessment dropout reasons:<br>▪ lack of time<br>▪ feeling too depressed<br>▪ slow or unreliable Internet connection<br>▪ intervention contained too much text<br>▪ CBT was too complicated to understand
[6] Gerhards, 2011 (NL)	Colour your life: online CBT self help program for depression.<br>Duration: 9 weeks<br>Target group: General public; mild to moderate symptoms	Qualitative study by means of patient (user) interviews	From thematic analyses barriers and motivators emerged	Important barriers are:<br>▪ Lack of identification with the program<br>▪ Lack of support (for discipline)<br>▪ Inadequate computer/internet skills<br>▪ No access to equipment<br>▪ Location of the computer (no privacy)
[7] Hedman, 2012 (SWE)	Cognitive behavioral therapy for SAD in group sessions	Regression analysis	Predictors and moderators; demographic, therapy-	Better treatment response in case of:<br>▪ working full time

	Duration: 15 weeks<br>Target group: patients in primary care		process related, genetic and clinical.	▪ having children<br>▪ less depressive symptoms<br>▪ higher expectancy of treatment effectiveness
[8] Kelders, 2013 (NL)	Voluit leven(living to the full): online ACT self-help program for despression with email support.<br>Duration: 9 weeks<br>Target group: general public; mild to moderate symptoms	Correlation analysis<br>Logistic regression<br><br>Log data analysis of use data	▪ Demographic variables<br>▪ need for cognition<br>▪ need to belong<br>▪ internet usage<br>▪ internet experience<br>▪ use during intervention (log data)	Adherers used the internet more often than non-adherers, were most often female, had a higher Need for cognition. Adherers logged on more often and spent more time loggen on per session.
[9] Neil, 2009 (AUS)	MoodGYM: self-help program for fear and depression based on CBT; self-directed program with monitoring/teacher support (in classroom)<br>Duration: 5 weeks<br>Target group: community adolescents	Linear regression analysis and t-tests to measure differences between support/no support.	Demographic variables<br>Severity of depression<br>Setting (with/without classroom support)	Adherers were more often enrolled in the school-based setting (monitored), were more often female and had severer levels of depression. In the condition without monitoring motivation was important to reach treatment effect.
[10] Nordgreen, 2012 (NOR)	Self-help for SAD	Comparisons between guided self-help & unguided self-help by means of t-test en logistic regression	Pretreatment symptoms<br>Program factors	'Credibility' of the intervention was associated with better adherence (more modules completed), both for guided and unguided)
[11] Spek, 2008 (NL)	Colour your life: group treatment vs. online self-help.<br>Duration: 9 weeks<br>Target group: General public; sub-threshold depression	Predictors of therapy outcomes by means of ANCOVA	▪ BDI scores<br>▪ Demographics<br>▪ Five main personality characteristics (Openness, Conscientiousness, Extraversion, Agreeableness, Neuroticism)	Neuroticisme is associated with worse outcomes in CCBT

[12] Vangberg, 2012 (NOR)	MoodGYM: prevention and treatment of depression; self-directed without support<br>Duration: 5 weeks<br>Target group: high school students	Regression analysis	▪ temperament = novelty seeking, harm avoidance, reward dependence, persistence<br>▪ character = self-directedness, cooperativeness, self-transcendence<br>▪ self-efficacy<br>▪ gender<br>▪ CES-D	Predicting outcomes for use were CES-D (mate van depressive) and ‘reward dependence’
[13] Wojtowicz, 2013 (CAN)	A guided online anxiety, depression, and stress self-help program for university students	Multiple regression analysis	▪ Age<br>▪ Symptom severity<br>▪ Beliefs and attitudes (TPB)	Perceived behavioural control and age predict the amount of finished modules.

Table 2. Systematic reviews on use/adherence/acceptance of CCBT via eHealth for depression and anxiety.

Artikel	Focus	Outcome measurements	Results	Conclusions
[14] Christensen, 2009 (AUS)	Adherence of self-help CCBT interventions (open access) for anxiety and depression	▪ Drop-out<br>▪ Compliance<br>▪ Predictors of adherence	▪ Increased adherence: lower baseline depression, younger age, poorer knowledge of treatment.<br>▪ Reasons for drop-out: time constraints, lack of motivation, technical problems, lack of face-to-face contact, perceived lack of effect, burden of the program	Little is known about factors that improve adherence. Studies differ too much in their approach to allow for comparisons and web-based data collection should be used more often.
[15] Kaltenhaler, 2008 (UK)	Acceptability of CCBT interventions for patients with mild to moderate depression	▪ Recruitment rates<br>▪ Drop-outs<br>▪ Reported acceptability/satisfaction	▪ Recruitment data is of limited worth, because it often concerns self-selection.<br>▪ Drop-out rates varied from 0 to 75%, these figures are comparable to regular CBT. Calculation of drop-out differs and reasons for drop-out are supplied scarcely.<br>▪ Data on acceptability are only available for participants who completed the intervention, these participants mostly rate acceptability positively	Studies differ strongly regarding design, study population, recruitment and content of the intervention. Little information is given on drop-out and satisfaction. Research into initial engagement, continuation and satisfaction is needed.
[16] Waller, 2009 (UK)	Barriers to uptake of CCBT for anxiety and depression	▪ Acceptability<br>▪ Accessibility	▪ Reasons for drop-out: lack of time, 'therapy'(but unclear whether it applies to participants in cCBT, control, or both)<br>▪ Satisfaction in qualitative studies is often high. Negative experiences include: too demanding, patronizing, fast-paced, computer was 'cold'<br>▪ Accessibility was related to computer literacy (experience)	Studies zijn erg heterogeen in focus, methodologie en kwaliteit. Kwaliatieve data is van belang. De belanrijkste barrières lijken computer/internet toegang, tijdsinvestering en computer/internet vaardigheden.
[17] Or, 2008 (Hong Kong & US)	Patient acceptance of self-care or primary care services	Variables for Health Information Technology acceptance	▪ Broad search, including e.g., informative websites, decision aids, and e-consult.<br>▪ 94 individual factors found, related to the individual (=71% of all identified factors; socio-demographics, skills,	The authors plead for a holistic approach to the development, implementation, and evaluation of eHealth interventions, with a stronger focus on the system and the

			experience), other factors are related to human-technology interaction, organizational and environmental factors.<br>▪ Studies give little theoretical support for their study goals. Therefore, meta-analyses are difficult to perform<br>▪ Demographic variables showed no or inconsistent effects (they are possibly moderators for computer literacy, fear, or experience)<br>▪ Computer- experience and human-technology interaction is predictive for acceptation	organization.<br>TAM, perceived usefullness and ease of use should be applied more often in studies, as well as computer self-efficacy.<br>Environmental factors and social factors can play a role as well (privacy, quiet place to work, social support (UTAUT)
[18]<br>Donkin, 2011 (AUS)	Adherence to e-therapy	▪ Adherence<br>▪ Effect of adherence on effectiveness	▪ Way of measuring adherence differs<br>▪ Regarding depression: completion of modules and general 'website exposure' was related to better outcomes	It seems that interaction with the system improves the treatment effect, not solely the use of the system. This is an important nuance.

References

1. Batterham PJ, et al., Predictors of adherence among community users of a cognitive behavior therapy website. Patient preference and adherence 2008; 2: 97. PMCID: PMC2770409
2. Bendelin N, et al., Experiences of guided Internet-based cognitive-behavioural treatment for depression: a qualitative study. BMC psychiatry 2011; 11(1): 107. PMID: 21718523
3. Cavanagh K, et al., The acceptability of computer-aided cognitive behavioural therapy: a pragmatic study. Cognitive behaviour therapy 2009; 38(4): 235-246. DOI:10.1080/16506070802561256
4. Donkin L, and Glozier N, Motivators and motivations to persist with online psychological interventions: a qualitative study of treatment completers. Journal of medical Internet research 2012; 14(3):e91. PMID: 22743581
5. Farrer LM, et al., Predictors of Adherence and Outcome in Internet-Based Cognitive Behavior Therapy Delivered in a Telephone Counseling Setting. Cognitive therapy and research 2014; 38(3): 358-367. DOI 10.1007/s10608-013-9589-1
6. Gerhards S, et al., Improving adherence and effectiveness of computerised cognitive behavioural therapy without support for depression: a qualitative study on patient experiences. Journal of affective disorders 2011; 129(1): 117-125. DOI 10.1016/j.jad.2010.09.012

7. Hedman E, et al., Clinical and genetic outcome determinants of Internet-and group-based cognitive behavior therapy for social anxiety disorder. Acta Psychiatrica Scandinavica 2012; 126(2): 126-136. DOI 10.1111/j.1600-0447.2012.01834.x
8. Kelders SM, Bohlmeijer ET, & van Gemert-Pijnen JEWC, Participants, usage, and use patterns of a web-based intervention for the prevention of depression within a randomized controlled trial. Journal of medical Internet research 2013; 15(8), e172. DOI 10.2196/jmir.2258. PMCID: 3757912
9. Neil AL, et al., Predictors of adherence by adolescents to a cognitive behavior therapy website in school and community-based settings. Journal of Medical Internet Research 2009; 11(1): e6. PMID: 19275982
10. Nordgreen T, et al., Outcome predictors in guided and unguided self-help for social anxiety disorder. Behaviour research and therapy 2012; 50(1): 13-21. DOI 10.1016/j.brat.2011.10.009
11. Spek V, et al., Predictors of outcome of group and internet-based cognitive behavior therapy. Journal of affective disorders 2008; 105(1): 137-145. DOI 10.1016/j.jad.2007.05.001
12. Vangberg HCB, et al., Does personality predict depression and use of an internet-based intervention for depression among adolescents? Depression research and treatment 2012; 2012. doi:10.1155/2012/593068
13. Wojtowicz M, Day V, McGrath PJ, Predictors of participant retention in a guided online self-help program for university students: prospective cohort study. Journal of medical Internet research 2013; 15(5): e96. DOI 10.2196/jmir.2323. PMID: 23697614
14. Christensen H, Griffiths KM, Farrer L, Adherence in Internet interventions for anxiety and depression: systematic review. J Med Internet Res 2009; 11(2): e13. DOI 10.2196/jmir.1194. PMID: 19403466
15. Kaltenthaler E, et al., The acceptability to patients of computerized cognitive behaviour therapy for depression: a systematic review. Psychological medicine 2008; 38(11): 1521-1530. DOI 10.1017/S0033291707002607
16. Waller R, Gilbody S, Barriers to the uptake of computerized cognitive behavioural therapy: a systematic review of the quantitative and qualitative evidence. Psychological medicine 2009;39(5): 705. DOI 10.1017/S0033291708004224
17. Or CK, Karsh B-T, A systematic review of patient acceptance of consumer health information technology. Journal of the American Medical Informatics Association 2009; 16(4): 550-560. DOI 10.1197/jamia.M2888. PMCID: 19390112
18. Donkin L, et al., A systematic review of the impact of adherence on the effectiveness of e-therapies. Journal of medical Internet research 2011; 13(3); e52. DOI 10.2196/jmir.1772. PMCID: 3222162
