# Supplementary material for: Mixing Online and Face-to-Face Therapy: How to Benefit From Blended Care in Mental Health Care
Source: JMIR Ment Health. 2016 Feb 9;3(1):e9. doi: 10.2196/mental.4534 (PMC4764785; doi:10.2196/mental.4534)
Supplement: Multimedia Appendix 2 [file mental_v3i1e9_app2.pdf]

Part	Item	Rationale
1. Prerequisites	1 Therapist checks: are appropriate online modules available related to the main symptoms/diagnosis of the patient?	Modules/ways of providing a certain type of care at a distance should be available to enable blended care.
	2 Therapist checks: Is there absence of (current) crisis (eg, severe suicidality or psychotic symptoms)?	If the patient is in a current crisis or problems are so severe that (s)he cannot work independently on his/her treatment (from home), blended care cannot be started, to warrant patient safety and treatment effect
	3 Therapist checks: Is there absence of an acute medical care need (that may hinder the patient's ability to independently work on his/her treatment?)	If severe medical problems prevent the patient from independently working on his/her treatment, blended care cannot be started, to warrant patient safety and treatment effect
	4 Therapist checks: Is the patient's intelligence quotient match sufficient for the blended treatment content?	Planned online activities/treatment modules should match the patients' cognitive abilities to warrant treatment effect.
	5 Patient checks: Does he/she have computer access?	Because blended care implies (a part of) the treatment will be computer mediated, having access to a computer is a prerequisite. This does not have to be the patient's own computer, as long as the environment (eg, a friend's house) offers sufficient privacy and trust.
	6 Patient checks: Does he/she have Internet access?	The patient needs Internet access to be able to follow online treatment. Again, the Internet connection of a trusted near

		one or secured public place might also suffice.
	7 Patient checks: Does he/she have a private, safe place to work?	The patient needs to feel secure (s)he can work in peace and with sufficient privacy on his/her treatment to warrant treatment adherence.
	8 Patient checks: Does he/she have sufficient Internet skills?	The patient needs to feel sufficiently secure (s)he masters the necessary skills that are needed to do the selected treatment modules independently. What skills are necessary depends on the specific modules or applications that will be used. A basic assessment can be made by asking about regular activities the patient performs online (eg, check email, buy something online)
	9 Patient and therapist discussions: Does the patient have sufficient writing (expression) skills?	In online care, patients often express themselves and communicate with the therapist in an asynchronous way. This means that there is no direct connection and real-time conversation. The therapist cannot probe or ask clarifying questions. So, to communicate effectively, patients need to be able to express themselves and their feelings in writing.
2. Possible barriers	1 Patient and therapist discussions: The patient's motivation and trust	The patient needs to be motivated to receive blended treatment and trust this treatment modality, to warrant the adherence to and effect of the treatment.

	2 Patient and therapist discussions: The patient’s risk of crisis	If a patient is in crisis, working independently on his/her treatment will become very difficult. If a crisis is foreseeable, this can be a barrier to effective blended treatment.
	3 Patient and therapist discussions: Cognitive problems that may hinder treatment	If the patient has cognitive problems (eg, attention deficits) that hinder working on the treatment independently, this can be a barrier for blended treatment.
	4 Patient and therapist discussions: Psychosocial problems that may hinder treatment	Psychosocial problems a patient has (severe financial issues, family issues) may interfere with the patient’s ability to take the time and focus to work on his/her treatment independently.
	5 Patient and therapist discussions: Other issues/comorbidity that may hinder treatment	The patient can have other issues or comorbidity (for which s/he does not receive treatment) that prevents her/him from working on her/his treatment independently due to a lack of focus.
3. Possible facilitators	1 Patient and therapist discussions: Whether they have (or chances on having) a good therapeutic/working relation	Having a good therapeutic/working relation is important to ensure trust between one another which will effect adherence and treatment success.
	2 Patient and therapist discussions: Possible practical reasons for preferring blended care, such as saving on cost and time of travel to the clinic, comfort.	Patients may prefer to undergo part of their treatment independently, because it saves them the practical hassles associated with visiting the therapist in person.

	3 Patient and therapist discussions: Possible other reasons for preferring blended care such as stigma or safety issues (shame of having to enter a clinic, discussing reason for taking time off work with employer, fears of going out into public to travel to a clinic)	Some patients may experience anxiety or fear to go out to a therapist, or are fearful of other persons finding out they go to the therapist's office. Although giving in to (irrational) fears is not a strategy blended care aims to support, blended care does have the ability to lower the barrier of seeking therapist help for persons who suffer from these issues.
	4 Patient and therapist discussions: The likeliness of being able to be open in online communication	Openness in online communication is key for successful online treatment due to the large use of written messages, or other synchronous or asynchronous communication without nonverbal ques. For some patients, taking about difficult (eg, emotional or shame associated) topics can be easier in non–face-to-face settings. Furthermore, patients have time to reflect on their messages before sending them, offering important insights without therapist intervention.
	5 Patient and therapist discussions: Is the patient conscientious?	A patient who knows (s)he is conscientious can benefit from a more efficient treatment setup via blended care, receiving the trust and support to undergo part of the treatment in a more independent fashion.

	6 Patient and therapist discussions: Does the patient have a social support network?	When patients are supported by their family or near friends to work on their treatment, the "burden" of working on the treatment independently is lowered.
4. Advice overview	1 Patient and therapist reach agreements based on: Overview of barriers and facilitators, including advice on how to cope with or profit from them in treatment	Barriers that are present for a patient are not necessarily a reason not to start blended care. Likewise, facilitators do not lead to guaranteed blended treatment success. Rather, patients and therapist can discuss how to deal with barriers and use the facilitators to their advantage if blended treatment is started.
	2 Patient and therapist reach agreements based on: Advice on blend (amount of online vs face-to-face)	The patient and the therapist discuss, based on the discussion of barriers and facilitators, that is, the likely combination of online and face-to-face contact they will have. This does not have to be 50:50.
	3 Therapist considers: Advice for therapist regarding treatment content	The therapist, knowing which modules or eHealth modalities are available (eg, treatment components, text messages, or video-calling support) proposes which parts of treatment can be confidently done via an online treatment platform and which ones will be done in face-to-face settings.
